# A Case of Diphtheria Successfully Relieved by Laryngotomy

**Published:** 1874-02-15

**Authors:** H. A. Johnson


### A CASE OF DIPHTHERIA SUCCESSFULLY RELIEVED BY LARYNGOTOMY.

REPORTED BY H. A. JOHNSON, M.D.

ALICE Wrenn, aged three and one-half years, had been in poor health during the summer of 1873; was sent to the seashore; did not improve there; was taken into the mountains of Eastern New York, where she improved materially; came home about the 1st of October, 1873, in pretty good health. On the 17th of November, she began to complain of headache, and had fever. She was treated for "sore throat," by a homeopathic physician, from November 17th to November 27th. The mother and father state that she had a coating of whitish matter over the back part of the mouth. She had some difficulty in swallowing, during this time, and some swelling of the glands of the neck; and, on the evening of the 26th, began to be hoarse. On the 27th, at 10 P. M., I saw her, with Dr. H. N. Hurlbut, who had been called to attend the case. She had, then, great difficulty of breathing, both of inspiration and expiration; voice nearly extinct; patches of diphtheritic membrane were attached to the fauces and soft palate; the portions not covered were of a dark, livid color; pulse small and frequent; temperature, in the axilla, 98½°; She was ordered sat. solution of potass. chlorate, with muriatic acid and stimulants.

*Nov.* 28*th*, 8 A. M.—Breathing still more oppressed; no improvement in any respect; pulse very weak and small; skin cooler and moist; no moist rales in the chest; voice, entirely extinct. At 9.30, Professor M. Gunn, who had been called in the morning, performed laryngotomy. There was considerable loss of blood, but she rallied; and a tube was inserted into the opening. Breathing soon became easy and free. A steam atomizer, with a solution of potass. chlorate, was kept in operation near the bed; and the temperature of the room was maintained at 80° F

*Nov.* 29*th*, A. M.—Some obstruction of the tube; removed it, cleansed it, and re-inserted it. Evening: some cough; had a little fever last night, and has a little to-night; has taken, since the operation, freely of milk; no medicine, except the solution of potass. chlorate, with the atomizer; sits up in bed and drinks.

*Nov.* 30*th*, A. M.—Has frequent paroxysms of coughing, with expectoration of dark, purulent matter; still takes milk; no bronchial trouble; the tube has been removed several times.

*Dec* 1*st*.—Expectoration through tube, liquid, bloody; more free than yesterday, still dark; takes food; atomizer kept in action, with water instead of the potass. chlorate solution, half of the time; had a bad time last night.

*Dec.* 7*th*.—She has been constantly gaining in strength; desires and takes solid food; cannot yet breathe through the glottis; in swallowing liquids, some portion of them pass out by the side of the tube—this is accompanied with paroxysms of coughing; she sits up, and wants to be dressed; plays with her dolls and pictures; expectoration mucous, with occasionally small streaks of muco-purulent matter. The mother and grandmother have for several days had sore throat, with swelling of the glands of the neck, and difficulty in swallowing.

*Dec. 11th.*—She had, at twelve o'clock last night, a severe spasm of coughing, with spasm of the thoracic muscles. This seemed to prostrate her very much. In the morning, removed the tube; she breathes quite easily through the nose and mouth; took her breakfast well; fluids still tickle the larynx—a few drops appearing at the opening made by the operation.

*Dec. 14th.*—She has been steadily gaining strength; appetite good; coughs less; expectoration mucous; is still troubled in swallowing liquids; no difficulty with solids; has gained in strength, and apparently in flesh; breathes almost entirely through the natural passages; the opening at the point of operation very much contracted.

*Dec. 18th.*—Opening completely closed; coughs but little; drinks better, but still some spasm of the glottis, unless the quantity of fluids is very small, and the act of swallowing performed slowly; speaks still in a whisper, but occasionally a slight sound is made; appetite good; amuses herself with her toys.

*Dec. 25th.*—Speaks aloud with ease, but the voice is a little husky; limbs weak.

*Jan. 7th*, 1874.—Is gaining in strength; voice good; recovery complete. Mother and grandmother, and child with grandmother, had diphtheria.

